# An exploratory and confirmatory analysis of the oxford hip score: generation of subscales assessing self-reported function and pain

**DOI:** 10.1186/1745-6215-14-S1-P74

**Published:** 2013-11-29

**Authors:** Kristina Harris, David Beard, Jill Dawson, Andrew Price

**Affiliations:** 1 University of Oxford, Oxford, UK

## Aims

The aim of this study was to explore dimensionality of the Oxford Hip Score (OHS) and examine if pain and self-reported functioning can be distinguished from the OHS in the form of subscales.

## Methods

Secondary data analysis of the UK National Health Service (NHS) audit dataset containing pre-operative OHS scores on 134, 710 patients who were undergoing hip replacement surgery.

## Results

The proposed number of factors to extract depended on the method employed. Velicer’s Minimum Average Partial test suggested one factor, the Cattell’s scree test and parallel analysis suggested two factors, and the second eigenvalue of 1.04 also suggested two factors. Exploratory factor analysis demonstrated that the 2-factor OHS had most of the items saliently loading either of the two factors. These factors were named ‘Pain’ and ‘Function’ and their respective subscales were created. Cronbach’s alpha was 0.90 for the 12-item summary scale, 0.85 for the 6-item ‘Function’ subscale, and 0.82 for the 6-item ‘Pain’ subscale. Confirmatory factor analysis demonstrated that the 2 factor model of the OHS had somewhat better fit. However, none of the one factor or two factor models was rejected.

## Conclusions

Factor analyses demonstrated that, in addition to the current use a single summary scale, separate information on pain and self-reported function can be extracted from the OHS in a meaningful way, in the form of subscales.

